# Predictors of significant distress in cervical cancer patients: a cross sectional study

**DOI:** 10.1007/s00404-024-07505-2

**Published:** 2024-04-23

**Authors:** Friederike Schmitt, Laila Najjari, Tomas Kupec, Elmar Stickeler, Ivo Meinhold-Heerlein, Julia Wittenborn

**Affiliations:** 1 Department of Gynecology and Obstetrics, University Hospital of the RWTH Aachen, Pauwelsstraße 30, 52074 Aachen, Germany; 2 Department of Gynecology and Obstetrics, University Hospital Gießen, Justus Liebig University, Klinikstraße 33, 35392 Gießen, Germany

**Keywords:** Cervical cancer, NCCN distress thermometer, Peritherapeutic distress, Psychooncology

## Abstract

**Purpose:**

This cross-sectional study aims to investigate parameters that predict relevant levels of distress in women in a perioperative setting undergoing treatment for cervical cancer.

**Materials and methods:**

Data from 495 patients with cervical cancer that were treated at the university hospital Aachen between 2010 and 2022 were analysed based on their respective National Comprehensive Cancer Network (NCCN) Distress Thermometer score (DT) and Problem List (PL) and their clinical history. 105 patients were enrolled in the study. 18 medical and demographic variables were analysed using multivariate logistic regression.

**Results:**

Three variables contributed significantly to the prediction of a DT score ≥ 5. Significant distress was defined as a DT score of ≥ 5, which was observed in 70.5% of the participants (mean: 5.58 ± 2.892). Women who chose to receive psycho-oncological counselling were more likely to have a DT score ≥ 5 (Odds Ratio(OR) = 3.323; Confidence Interval (CI95%): 1.241–8.900; p-value: 0.017). In addition, women who did not receive chemoradiation had significantly higher DT scores (OR = 3.807; CI 95%:1.185–12.236; p-value: 0.025), as did women whose Distress Thermometer was assessed in the first month after their initial diagnosis (OR = 3.967; CI 95%:1.167–13.486; p-value: 0.027).

**Conclusion:**

Increased distress in women with cervical cancer is common especially in the first month after diagnosis, in patients who do not receive chemoradiation and in patients who seek psycho-oncological counselling. Surgical factors do not play a major role in patient distress.

## What does this study add to clinical work

**Table:** 

A large percentage of cervical cancer patients have increased levels of distress and are a highly vulnerable group of patients. This study is the first to specifically examine the factors predicting distress in cervical cancer patients in a perioperative setting. The following parameters predictited a DT score ≥ 5 significantly: patients choosing to receive psycho-oncological counselling, patients with no chemoradiation and those in the first month after their initial diagnosis.	

## Introduction

Cervical cancer is the fourth most common malignancy in women worldwide [1]. With the introduction of cervical cancer screening programs, a decrease in the incidence of cervical cancer since 1970 was achieved. Nonetheless 4575 women were newly diagnosed and 1597 died from cervical cancer in Germany in 2019 [2]. The process of the diagnosis of cervical cancer alone is distressing [3] and being diagnosed is a life-changing event and often causes suffering [4]. This is illustrated by the fact that significant distress has been reported in up to 63.1% of women with gynaecological cancer [5–8].

Distress is more than an emotional symptom, it is a complex phenomenon: The National Comprehensive Cancer Network (NCCN) defines it as a “multifactorial, unpleasant experience of a psychological, social, spiritual and or physical nature that may interfere with the ability to cope effectively with cancer, its physical symptoms and its treatment” [9]. Its evaluation in oncology patients is part of the clinical routine, in adherence to NCCN recommendations, which state that distress should be assessed at every visit [9, 10].

The Distress Thermometer (DT) and Problem List (PL) are a brief screening tool to identify and address psychosocial distress in oncology patients. Translations are available in 71 languages. They have been validated internationally and are also recommended in the German S3 guidelines for psychooncology [11, 12].

There is much debate about whether laparoscopic or laparotomic surgery is the better approach for patients with cervical cancer. In 2018 Ramirez et al. showed that laparotomy was associated with better 4.5 year disease-free survival [13]. Despite this discussion, the influence of treatment options on peritherapeutic distress of patients has yet to be considered. Several studies have shown that cancer patients undergoing treatment generally suffer from high levels of distress [14, 15]. In addition, distress can influence the progression and outcome of many types of cancer as it is affecting patient compliance and treatment [16–18]. If left untreated, distress can lead to longer hospital admissions and higher amounts of pain, which can negatively impact the patients as well as the health care system [19–22]. It has also been found that screening for distress may lead to earlier referral to a support service [23]. All these findings underline the utmost importance of assessing distress in oncologic patients at an early stage and to identify patients at special risk.

We present the results of our cross-sectional study about distress of women undergoing treatment for cervical cancer at the Department of Gynaecology and Obstetrics at the university hospital Aachen. This study aimed to investigate whether specific characteristics of patients with cervical cancer predict elevated distress levels, offering implications for clinical management in order to avoid negative secondary effects for the patient caused by the distress. We chose to explore surgical, general therapeutic, personal as well as demographic factors in the analysis. It was hypothesized that cervical cancer patients undergoing treatment show elevated distress levels and sought to find the key influencing factors in order to prioritise patients at risk for early psychological support.

## Materials and methods

### Study design and population

This cross-sectional study was carried out at the university hospital in Aachen, Germany. All patients who were initially diagnosed with cervical cancer and treated at the Gynaecological Cancer Unit of the Department of Gynaecology and Obstetrics at the RWTH university hospital between 2010 and 2022 were assessed for eligibility, totalling 495 female patients. All identified patients were retrospectively analysed. Sample size was determined by including all cases of cervical cancer and subsequently excluding all patients who did not meet our criteria aiming to avoid bias and specify our results.

Inclusion criteria for our study were the following: Patients were initially diagnosed and treated at the RWTH university hospital. Additionally, patients were required to complete the Distress Thermometer within 2 months after treatment for cervical cancer and within 6 months after their initial diagnosis, to ensure that the measured distress stems from the condition and its treatment.

Patients who did not complete the Distress Thermometer were excluded (n = 371).

The remaining 124 patients completed the Distress Thermometer in the Department of Obstetrics and Gynaecology, where they received surgery or in the Department of Radiooncology, where they received chemoradiation.

**Fig. 1:**
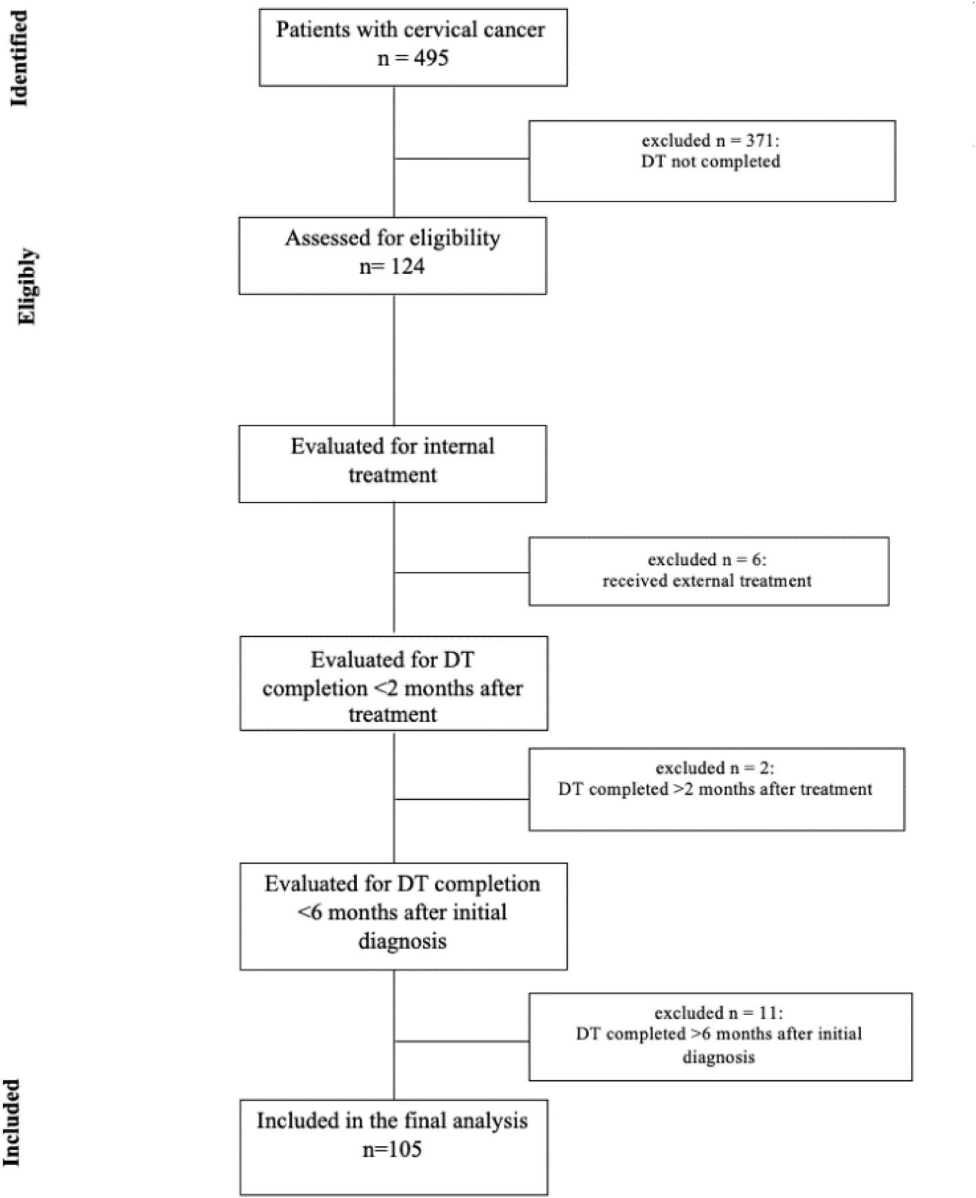
Consort flow diagram

In the end, 105 patients were included in the final analysis (Fig. 1).

### The NCCN distress thermometer

The Distress Thermometer was developed by the NCCN in the USA and first published in 1998 [24]. It is a self-administered instrument that uses an eleven-point scale ranging from 0 (no distress) to 10 (extreme distress), it is presented on a drawn thermometer. Patients are asked to rate their level of distress over the past week. The Distress Thermometer is accompanied by a Problem List of 41 dichotomous questions that aim to identify the causes of distress [10, 25]. It includes inquiries into family problems, emotional problems, practical problems, physical and spiritual problems. The NCCN also provides referral schemes to the appropriate professional support services, depending on the problem that has been identified.

The Distress Thermometer is a very sensitive tool that has been validated internationally [11, 14, 24, 26, 27]. For validation the Hospital Anxiety Depression Scale (HADS), the General Health Questionnarie-12 (GHQ-12) or the Brief Symptom Interventionary-18 (BSI-18) were used [28].

We chose a cut-off score of 5 according to the guidelines of the German society of psychooncology [29, 30].

### Data collection

Patient data was recorded as shown in Table 1.

**Table 1:** Patient baseline characteristics

Variable	Total	DT < 5(n)	DT < 5(%)	DT ≥ 5(n)	DT ≥ 5(%)
n =	105	31		74	
Age (in years)					
Mean		51.81		50.18	
Standard deviation (SD)		13.821		13.458	
Median		53		51.5	
Interquartile range (IQR)		23		22.5	
Surgery duration (in min)					
Mean		271.32		274	
SD		127.07		139.138	
Median		283		256	
IQR		185		185	
Neoadjuvant chemotherapy					
Yes	6	1	3.2	5	6.8
No	99	30	96.8	69	93.2
Peri-dural catheter					
Yes	78	26	83.9	52	70.3
No	27	5	16.1	22	29.7
Partnership^a^					
Yes	55	17	54.8	38	51.4
No	50	14	45.2	36	44.6
Chemoradiation					
Yes	64	23	74.2	41	55.4
No	41	8	25.8	33	44.6
Surgery^b^					
Diagnostic	64	18	58.1	46	62.2
Therapeutic	41	13	41.9	28	37.8
Surgical technique					
Laparotomy	13	4	12.9	9	12.2
Laparoscopy	78	24	77.4	54	73
Other	14	3	9.7	11	14.9
FIGO stage					
≤ I	50	16	51.6	34	45.9
> I	55	15	48.4	40	54.1
Complications^c^					
≤ 1	86	26	83.9	60	81.1
> 1	19	5	16.1	14	18.9
Removed pelvic lymph-nodes					
n < 20	51	12	38.7	39	52.7
n ≥ 20	54	19	61.3	35	47.3
Pelvic lymph-nodes involved					
Yes	27	10	32.3	17	23
No	78	21	67.7	57	77
Removed paraaortic lymph- nodes					
n < 12	78	24	77.4	54	73
n ≥ 12	27	7	22.6	20	27
Paraaortic lymph-nodes involved					
Yes	15	5	16.1	10	13.5
No	90	26	83.9	64	86.5
Time between first diagnosis & DT assessment					
≤ 1 month	87	22	71	65	87.7
> 1 month	18	9	29	9	12.2
Stay in hospital					
≤ 1 week	48	17	54.8	31	41.9
> 1 week	57	14	45.2	43	58.1
Selection of a psycho-oncological counselling					
Yes	51	10	32.3	41	55.4
No	54	21	67.7	33	44.6
DT assessment in weeks after treatment					
≤ 2 weeks	93	28	90.3	65	87.8
> 2 weeks	12	3	9.7	9	12.2

Patient characteristic: All demonstrated patient characteristics are part of the final multivariate logistic regression model

^a^partnership = marriage or partner in life

^b^diagnostic = lymph-node staging or cystoscopy or rectoscopy, therapeutic = Wertheim surgery or hysterectomy or adnexectomy or salpingectomy

^c^complications = neurological or surgical or dermatological or internal or other

All patients included in the study were treated in the highly specialized Centre for Integrated Oncology (CIO), where multidisciplinary teams work together to optimize the care of oncological patients. At the Gynaecological Cancer Unit of the Department of Gynaecology and Obstetrics at the university hospital of Aachen patients are asked to complete the Distress Thermometer during an inpatient stay following their initial diagnosis of cervical cancer. Patients voluntarily complete the Distress Thermometer under supervision of a health care professional. Patients are made aware that psychological support is available, should they wish it.

The tumour stage is classified according to the International Federation of Gynaecology and Obstetrics (FIGO). The FIGO stage was determined from previous findings or classified according to the German S3 guideline “Therapy and follow-up care in patients with cervical cancer” using the medical history. The stages were categorized into the FIGO I stage and the stages above FIGO I [31].

Surgical procedures were divided into the categories “diagnostic intervention” or “therapeutic intervention”. Diagnostic intervention includes lymph node staging, cystoscopy and rectoscopy. If adnexectomy and salpingectomy were performed as part of the lymph node staging patients were allocated to the diagnostic intervention group. Therapeutic intervention includes Wertheim surgery, hysterectomy, adnexectomy and salpingectomy. Surgical technique was determined from the surgical reports and categorized as laparoscopy, laparotomy and other. Patients whose lymph nodes were extracted as part of the therapeutic surgery or who received a lymph node staging in advance and afterwards any type of therapeutic surgery were allocated in the therapeutic surgery group, as it was assumed that this would have the major impact on the patients distress. 22 patients underwent lymph node staging in addition to their therapeutic surgery, 19 received therapeutic surgery only. We included all patients in our lymph node count (paraaortic lymph nodes < 12/ ≥ 12, pelvic lymph nodes < 20/ ≥ 20) to investigate whether the number of extracted lymph nodes has an impact on the distress, independent of which kind of surgery they received.

We collected the data on complications from the previous medical history and classified them into the two categories: ≤ 1 complication and > 1 complication. Complications include, but are not limited to: surgical, internal, neurological and dermatologic.

According to the guidelines for early cervical cancer, the patients received unimodal treatment in order to reduce morbidity [31], wherever possible, as shown in Table 2.

**Table 2:** Diagnostic vs. therapeutic * chemoradiation cross tabulation

		Chemoradiation		Total
		No chemoradiation	Chemoradiation	
d.vs.t	Diagnostic intervention	14	50	64
	Therapeutic intervention	27	14	41
Total		41	64	105

### Statistical analysis

All statistical analysis was performed using IBM SPSS 29. Categorical variables were expressed as absolute frequencies and percentages. Continuous variables were tested for normal distribution and were expressed as median ± interquartile range (IQR) and mean ± standard deviation (SD).

The main goal of the analysis was to identify parameters predictive of significant peritherapeutic distress (DT score ≥ 5) in patients with cervical cancer. Multivariate binominal logistic regression was used to identify predictor variables from 18 independent variables and relationships between variables.

The dependent variable was the peritherapeutic distress. For all included patients, the dataset was complete. All 18 variables were included in the multivariate regression model. Stepwise backward (conditional) multivariate logistic regression was performed. Based on the conditional parameter estimates the probability likelihood ratio statistic was used to perform a removal testing.

The p-value for inclusion was 0.05 and the p-value for exclusion was 0.10.

P-values < 0.05 were considered statistically significant.

## Results

**Fig. 2:**
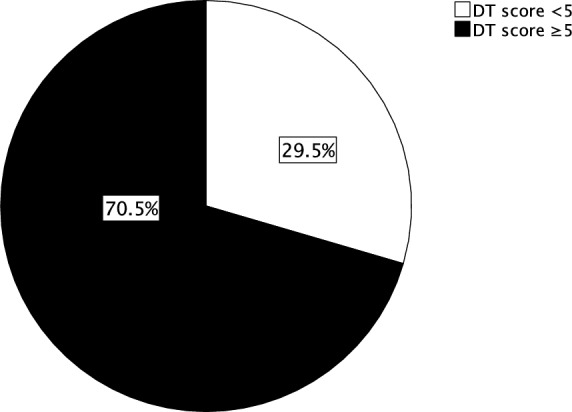
Distribution of DT score < 5/ ≥ 5 in percent

**Fig. 3:**
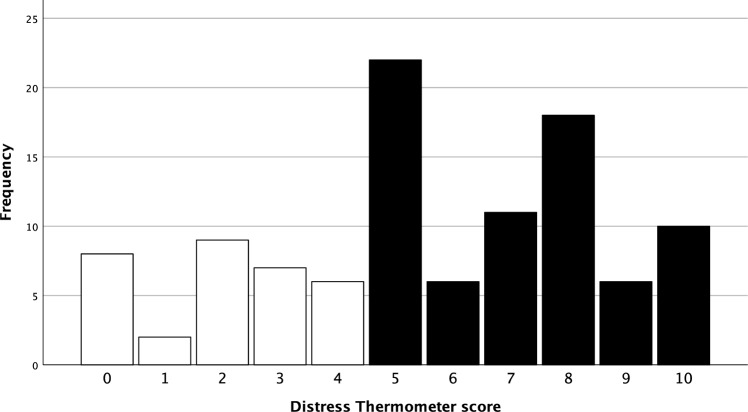
Frequency analysis and bar chart of the Distress Thermometer. DT scores ≥ 5 are colored in black, DT scores < 5 are colored in white. Cut-off score of 5 was chosen. The most common DT score was 5 (n = 22)

Patients baseline characteristics are shown in Table 1. All demonstrated patient characteristics are part of the final multivariate logistic regression model:

On average, the 105 patients were 51.81 (± 13.821) years old. All patients were diagnosed with cervical cancer at different stages, 50 patients had a FIGO stage I*.* 78 patients underwent laparoscopic surgery and 13 underwent laparotomy. More patients underwent diagnostic surgery (n = 64) than therapeutic surgery (n = 41). 64 patients received chemoradiation. A total of 51 patients chose to receive a psycho-oncological counselling during their treatment. Frequency analysis showed that 70.5% of the patients had a DT score ≥ 5 (Fig. 2) and that the mean DT score was 5.58 (± 2.892) (Table 3). Additionally, findings indicated that the most common DT score was 5 (Fig. 3).

**Table 3:** Distress thermometer statistics

DT		
N	Valid	105
	Missing	0
Mean		5.58
Median		5.00
Std. Deviation		2.892
Variance		8.361

Frequency analysis Distress Thermometer score absolute, mean, standard-deviation and variance of the Distress Thermometer as a continuous variable

Stepwise backward conditional multivariate logistic regression was performed. The final multivariate model was statistically significant with χ^2^ (6) = 21.761 and p < 0.01 (Table 4). The acceptable amount of explained variance was indicated by Nagelkerke´s *R*^2^ = 0,266. Goodness of fit was assessed using the Hosmer Lemeshow Test indicating a good model fit with χ^2^ (8) = 4.265, p > 0.05 [32, 33].The overall percentage of accuracy was 75.2% with a sensitivity of 87.8% and a specificity of 45.2%.

**Table 4:** Final multivariate logistic regression model

		B	S.E	Wald	df	Sig	Exp(B)	95% CI for EXP(B)	
								Lower	Upper
Step 13	Diagnostic vs. therapeutic(1)	− 1.114	.619	3.245	1	.072	.328	.098	1.103
	Peri-dural catheter(1)	− 1.158	.625	3.425	1	.064	.314	.092	1.071
	Stay in hospital (1)	1.083	.578	3.515	1	.061	2.953	.952	9.158
	Psycho-oncology counselling(1)	1.201	.503	5.710	1	**.017**	3.323	1.241	8.900
	Time DT assessment &initial diagnosis (1)	1.378	.624	4.872	1	**.027**	3.967	1.167	13.486
	Chemoradiation(1)	1.337	.596	5.037	1	**.025**	3.807	1.185	12.236
	Constant	− .482	.763	.400	1	.527	.617		

Significant values predicting a DT score ≥ 5 are indicated in bold. Variable(s) entered on step 1: age (years), FIGO stage (≤ I/ > I), diagnostic vs. therapeutic surgery, surgical technique(laparoscopic/laparotomy/other), neoadjuvant chemotherapy, lymph-nodes pelvin removed(< 20/ ≥ 20), lymph-nodes pelvin involved, lymph-nodes paraaortic removed (< 12/ ≥ 12), lymph-nodes paraaortic involved, peri-dural catheter, complications(≤ 1/ > 1), stay in hospital (≤ 1 week/ > 1 week), selection of a psycho-oncological counselling, time between DT assessment & initial diagnosis(≤ 1 month/ > 1 month), DT when evaluated (≤ 2 weeks after treatment/ > 2 weeks after treatment), chemoradiation, partnership, surgery duration(min)

Of the 18 variables included in the model the selection of a psycho-oncological counselling, chemoradiation and the time between the initial diagnosis and Distress Thermometer assessment were significant predictors of the dependent variable DT score ≥ 5. Patients who requested a psycho-oncological counselling were more likely to have a DT score ≥ 5 compared to patients who refused a psycho-oncological counselling OR = 3.323 (95%-CI [1.241; 8.9] p-value: 0.017). Chemoradiation and the time between the initial diagnosis and Distress Thermometer assessment had a protective effect on our outcome variable, reducing the likelihood of distress. The likelihood of having a DT score ≥ 5 without chemoradiation is increased compared to patients who received chemoradiation OR = 3.807 (95%-CI [1.185; 12.236] p-value: 0.025). Patients who completed the Distress Thermometer in the month following their initial diagnosis of cervical cancer were more likely to have a DT score ≥ 5 OR = 3.967 (95%-CI [1.167; 13.486] p-value: 0.027), compared to the patients who completed it later.

None of the other variables had a significant influence on our dependent variable DT score ≥ 5.

## Discussion

604,000 new cases and 342,000 deaths related to cervical cancer, a preventable disease, were reported globally in 2020 [1]. As such, it remains a public health issue and the subject of much debate. No previous studies investigated the risk factors that may predict distress in cervical cancer patients in a perioperative setting. In this study we present data from 105 cervical cancer patients, using the NCCN Distress Thermometer to measure the distress in these patients.

This study demonstrates that cervical cancer patients undergoing treatment have increased levels of distress. Three main predictors were found: We were able to show that patients who chose to receive a psycho-oncological counselling were 3.3 times more likely to have a DT score ≥ 5 than those who refused. Women who were not treated with chemoradiation were 3.8 times more likely to have a DT score ≥ 5 compared to women who received chemoradiation. The likelihood of a DT score ≥ 5 is 3.9 times higher when assessed in the first month after initial diagnosis of cervical cancer than at a later point. An accuracy of 75.2% was achieved.

The Distress Thermometer is a valid diagnostic tool, Gessler et al. investigated the validity of the Distress Thermometer and the Problem List relative to the widely accepted Hospital Anxiety and Depression Scale (HADS), the General Health Questionnaire 12 (GHQ-12) and the Brief Symptom Inventory 18 (BSI 18) and found the Distress Thermometer to be a valid and acceptable screening tool [28].

Compared to patients suffering from other cancer entities, the investigated patient group had remarkably high DT score values with a mean value of 5.58 (± 2.892). In patients with various types of cancer (gastrointestinal, gynaecological, pulmonary) the mean value was lower with reported DT scores of 3.9 (± 2.7) [34]. The relative number of patients with significant distress was also high in our study compared to other patients collectives. For example in a patient collective of brain cancer, significant distress (DT score ≥ 5) was found in 56.8% of the patients and in a collective of lung cancer it was 61.6%, whereas we found elevated distress in 70.5% of our patients. [35, 36].

These findings raise the question why the DT scores are elevated in the examined patient population. One possible explanation is that the patient population was all female and women are more likely to report higher distress scores, which has been shown previously in other types of cancer [7, 37, 38]. Some authors report that cervical cancer patients have a high prevalence of pain (84%) or vaginal discharge (66%), leading to anxiety (63%) and depressed mood (52%) [39], which may also cause significant distress. Women with gynaecological cancer have also been found to suffer from higher levels of loneliness than healthy women [40], and experience severe sexual dysfunction, including issues with orgasm or dyspareunia [41, 42], which can also lead to distress.

In order to elucidate the reasons for peritherapeutic distress in women with cervical cancer, we performed a mulitvariate analysis with a wide range of possible influencing factors including surgical, general therapeutic, personal as well as demographic factors in the analysis. We observed that patients who chose to receive psycho-oncological counselling are more likely to report DT scores ≥ 5. Patients who choose to receive psychological help are usually more distressed than those who did not. In our study the Distress Thermometer identified these patients reliably which demonstrates that the Distress Thermometer is a valid screening tool for patient distress in clinical practice.

As a second factor we found that a Distress Thermometer assessment in the first month after initial diagnosis of cervical cancer contributed significantly to predict DT scores ≥ 5. This is in line with the findings of Carlson et. al., who found that DT scores decreased in cancer patients over the time throughout therapy [43].

Interestingly we found chemoradiation to be a protective factor, significantly reducing the likelihood of a DT score ≥ 5. Jewett et al. describe chemotherapy as a risk factor for significantly high distress in women with cervical cancer [44]. Similarly, Li et al. found radiotherapy to be a risk factor of high distress in cervical cancer patients [45]. A possible explanation for the lower distress in patients with chemoradiation in our collective is that patients who receive chemoradiation frequently presented to the Department of Radiooncology and had more contact with their treating physician or oncological health care professionals. This possibly resulted in patients feeling more cared for which could reduce distress. In addition, patients who receive chemoradiation and not only therapeutic surgery are more involved in their own cancer treatment: They are for example awake during their therapy and are therefore more likely to understand what is happening to their own body. Because of this, patients receiving chemoradiation may have a greater sense of empowerment in the defeat of their cancer, which could explain the reduced likelihood of a DT score ≥ 5. Another possible explanation for the lower distress in patients with chemoradiation is that those patients had no operative and post-operative complications such as thrombosis, blood loss, urological injuries, or wound infections, which are very common after a radical hysterectomy surgery [46, 47]. It must also be noted that patients receiving chemoradiation experience fewer organ loss. Especially a hysterectomy can affect women’s body image and self-esteem [48] which may explain lower DT scores in a patient population that has not undergone therapeutic surgery.

Ramirez et al. showed that patients who underwent open surgery had a better 4.5 years disease free survival outcome than those who underwent laparoscopic surgery [13]. However, there is no data on whether laparotomic surgery is also better in terms of patient distress. We hypothezised, that patient distress in open surgery would be higher as it has been shown that laparoscopic surgery is associated with less intraoperative blood loss, shorter hospital stay and less postoperative morbidity [49]. Our study is the first one that explicitly evaluates a detailed list of operative factors in the analysis of peritherapeutic distress of cervical cancer. Interestingly, our findings do not indicate a significant influence of surgical technique on the outcome variable DT score ≥ 5. Patients who underwent laparoscopic surgery appeared to be equally distressed as patients who underwent laparotomic surgery. Other operative factors such as the number of pelvic and paraaortic lymph-nodes removed or the duration of surgery or postoperative complications also had no significant effect on a DT score ≥ 5.

Our study has strengths and limitations that need to be addressed. Our cross-sectional study includes a high number of patients with a full dataset and presents the first detailed analysis of a high number of potential operative influencing variables on patient distress in a cervical cancer cohort.

In terms of limitations, we performed a monocentric study which is a natural source of bias. Due to organisational challenges at the hospital, not all patients completed the DT. This lack in distress screening of inpatients in hospitals has been reported previously by the NCCN [50] and its improvement is part of our daily struggle for improvement of cancer patient care. Due to its random organisational cause, in our opinion, it is not a source of significant bias in the presented analysis. In order to establish causal relationships in the future, we intend to assess the distress thermometer longitudinally at different points of time during patient treatment.

## Conclusions

Our findings show that a high percentage of cervical cancer patients have increased levels of distress and are a highly vulnerable group of patients.

The study is the first to specifically examine the factors predicting distress in cervical cancer patients in a perioperative setting. The following parameters predictited a DT score ≥ 5 significantly: patients choosing to receive psycho-oncological counselling, patients with no chemoradiation and those in the first month after their initial diagnosis. This can help to better identify patients at risk in the future and prioritize them for psychological support enabling them to receive timely assitstance and interventions to adress their emotional and psychological needs. Operative variables such as surgical approach, the number of removed lymph-nodes or the duration of the surgery were not found to play a major role in patient distress.

In the context of early clinical management the emphasis should be placed on patients at risk of elevated levels of distress, as distinctly elucidated in our study, to avert negative consequences stemming from distress.

## Data Availability

The datasets generated in this study are available from the corresponding author upon request.
